# Effect of Macrolide Resistance and *Mycoplasma pneumoniae* DNA Load in Bronchoalveolar Lavage Fluid on Immune and Inflammatory Responses in Children with *Mycoplasma pneumoniae* Pneumonia

**DOI:** 10.1007/s13312-025-00190-7

**Published:** 2025-09-24

**Authors:** Haiqin Zhong, Zeyu Zeng, Haoxiang Gu, Xiaoyan Dong

**Affiliations:** Department of Respiratory Medicine, Shanghai Children’s Hospital, School of Medicine, Shanghai Jiao Tong University, No. 355, Luding Road, Putuo District, Shanghai, China

**Keywords:** Antibiotic resistance, *Mycoplasma pneumoniae* pneumonia, Pathogen load, Pediatric, Surveillance

## Abstract

**Objective:**

To investigate the impact of macrolide resistance and *Mycoplasma pneumoniae* DNA (MP-DNA) load in bronchoalveolar lavage fluid (BALF) on immune and inflammatory responses in children with *Mycoplasma pneumoniae* pneumonia (MPP).

**Methods:**

This retrospective study included 190 hospitalized children with MPP who underwent bronchoscopy. Patients were classified as macrolide-resistant or macrolide-sensitive based on 23S rRNA mutation analysis. MP-DNA load and cytokines in BALF and inflammatory markers in blood were measured. MRMP patients were further stratified by MP-DNA load for subgroup analysis.

**Results:**

Of 1029 children screened, 474 had MPP, and 190 who underwent bronchoscopy were analyzed. Macrolide-resistant mycoplasma pneumonia (MRMP) accounted for 73.2% of cases and was associated with longer fever duration and hospital stay, lower lymphocyte counts, higher BALF total cell counts and neutrophil proportions, and increased inflammatory cytokines. Among MRMP patients, those with high MP-DNA loads had greater BALF cytokine levels than those with low loads. MP-DNA load positively correlated with IL‑1β and IL‑6 levels.

**Conclusions:**

Macrolide resistance and high pathogen load are associated with enhanced airway inflammation and immune dysregulation in children with MPP. Early detection of resistance and quantification of pathogen load may guide timely antibiotic adjustment and improve clinical outcomes.

## Introduction

*Mycoplasma pneumoniae* (MP) is a leading cause of respiratory infections, especially among children and adolescents [1]. Although most infections are mild and self-limiting, some children develop severe or refractory disease requiring hospitalization [2, 3]. Macrolides remain the first-line antibiotics for *M. pneumoniae* pneumonia (MPP) in children due to their safety profile and efficacy [4]. However, the increasing prevalence of macrolide-resistant *M. pneumoniae* (MRMP) is a major clinical concern worldwide [5].

In Asia, MRMP prevalence in pediatric populations exceeds 80%, and infections are associated with delayed defervescence, prolonged hospital stay, and, in some cases, extrapulmonary complications [6]. Although large-scale data from India have been limited, recent molecular studies have confirmed the emergence of resistant strains. A recent report from Kerala identified the A2063G mutation in the 23S rRNA gene in 30.7% of clinical isolates, with a 40% macrolide treatment failure rate [7]. These findings highlight the growing importance of antimicrobial resistance surveillance and early detection strategies in children.

Pathogen load may also influence the disease severity and immune responses. Previous studies have suggested that high MP-DNA loads correlate with more severe clinical manifestations and stronger inflammatory responses [8]. However, few studies have comprehensively evaluated the combined impact of macrolide resistance and pathogen load on local and systemic immune responses in children with MPP.

This study aimed to investigate the clinical impact of macrolide resistance and bronchoalveolar lavage fluid (BALF) MP-DNA load on host immune and inflammatory responses in pediatric MPP.

## Methods

This retrospective study was conducted at Shanghai Children’s Hospital, Shanghai, China, and included children hospitalized with MPP between July 2022 and June 2023. The study was approved by the Ethics Committee of Shanghai Children’s Hospital. Written informed consent was obtained from the parents or legal guardians of all participants.

*Mycoplasma pneumoniae* pneumonia was diagnosed by DNA polymerase chain reaction (PCR) performed on the bronchoalveolar lavage fluid; patients underwent bronchoscopy and bronchoalveolar lavage (BAL) during the acute phase of illness (≤ 14 days from symptom onset). Children with bacterial or viral co-infections were excluded. No prior sample size calculation was performed because of the retrospective design.

Bronchoscopy was performed as part of the diagnostic evaluation in children with persistent fever (> 7 days), non-resolving pneumonia despite macrolide treatment, lobar consolidation on imaging, or suspected airway obstruction, following the Chinese Pediatric Flexible Bronchoscopy Guidelines [9]. BALF samples were immediately processed for cytological, molecular, and immunological analyses. BALF MP-DNA load was quantified using a commercial real-time PCR assay, and macrolide resistance was determined by sequencing the 23S rRNA gene V domain to detect A2063G mutations. Patients were classified as macrolide-resistant (MRMP) or macrolide-sensitive (MSMP). MRMP patients were further stratified by BALF MP-DNA load (> 10⁶ vs ≤ 10⁶ copies/mL).

Inflammatory cytokines (IL-1β, IL-5, IL-6, IL-8, IL-10, IL-17, IFN-γ, TNF-α) were measured in the BALF using multiplex bead-based immunoassays. Peripheral blood was analyzed for complete blood count, inflammatory markers [C-reactive protein (CRP), procalcitonin (PCT), erythrocyte sedimentation rate (ESR), D-dimer], liver enzymes, and immune cell subsets by flow cytometry.

*Statistical analysis*: Data were analyzed using SPSS 26.0. Continuous variables were presented as mean (standard deviation) or median (interquartile range) and compared using Student’s t-test or Mann–Whitney U test, as appropriate. Categorical variables were compared using the chi-square. Correlations between MP-DNA load and cytokines were analyzed using Spearman’s correlation coefficient. *P* < 0.05 was considered statistically significant.

## Results

**Fig. 1:**
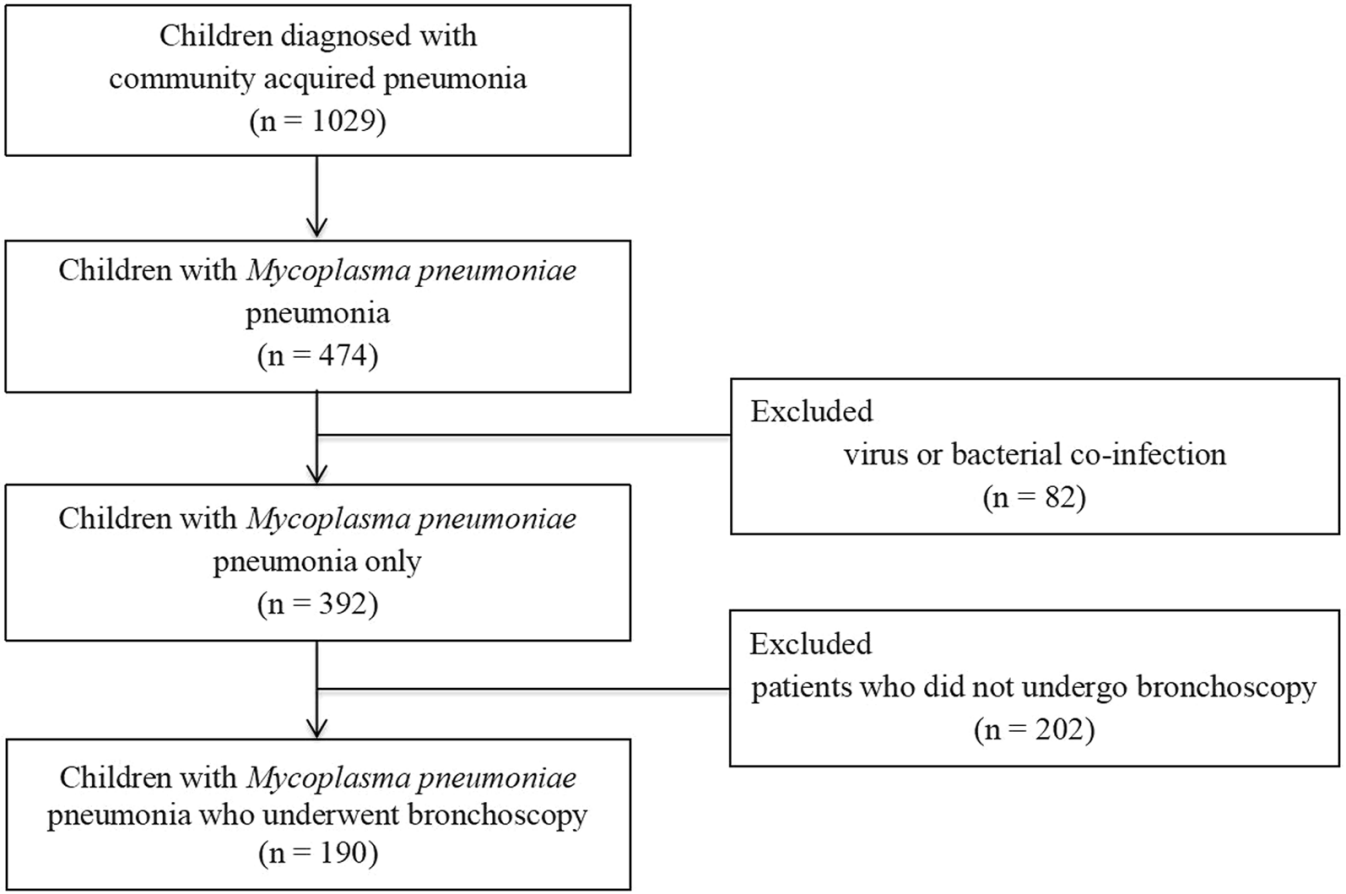
Study flow diagram of patient selection

During the study period, 1,029 children were hospitalized with community-acquired pneumonia, of whom 474 (46.1%) had MPP. After excluding 82 cases with viral or bacterial co-infections and 202 without bronchoscopy, 190 patients were included in the final analysis (Fig. 1).

Among the 190 patients, 139 (73.2%) were infected with MRMP and 51 (26.8%) with MSMP. Compared with MSMP, MRMP patients had significantly longer median duration of fever (6.0 vs 4.0 days) and hospital stay (7.0 vs 5.0 days), while other clinical features were similar between the two groups. Laboratory results showed that MRMP patients had lower white blood cell and platelet counts, reduced CD3 + , CD4 + , and CD8 + T-lymphocyte levels, and higher erythrocyte sedimentation rate (ESR), D-dimer, and IgA levels, but lower IgE levels. BALF analysis revealed higher total cell counts, an increased proportion of neutrophils and monocytes, and a lower proportion of macrophage and lymphocytes in MRMP compared with MSMP patients. Inflammatory cytokines, including IL-1β, IL-5, IL-6, IL-8, IL-10, IL-17, IFN-γ, and TNF-α, were all significantly elevated in the MRMP group (all *P* < 0.05). A detailed summary of these findings is shown in Table 1.

**Table 1:** Clinical characteristics and laboratory findings of MRMP and MSMP groups

Variable	Normal range	MRMP group (n = 139)	MSMP group (n = 51)	*P* value
*Demographics and clinical features*				
Age, years^a^	–	7.0 (6.0–8.0)	7.0 (5.0–8.0)	0.194^c^
Boys^b^	–	69 (49.6)	23 (45.1)	0.579^e^
Length of hospital stay, days^a^	–	7 (5–8)	5 (4–7)	0.001^c^
Fever duration, days^a^	–	6 (4–7)	4 (1–6)	0.005^c^
*Peripheral blood markers*				
WBC (*10⁹/L)^a^	4.0–10.0	6.3 (5.2–7.5)	7.9 (5.9–13.2)	< 0.001^c^
PLT (*10⁹/L)^a^	100–300	267.5 (223.0–323.0)	320.0 (228.0–401.0)	0.011^c^
PCT (ng/mL)^a^	< 0.1	0.1 (0.1–0.2)	0.1 (0.0–0.2)	0.531^c^
ESR (mm/h)^a^	0–20	41.0 (28.0–58.0)	29.0 (12.0–47.0)	0.017^c^
CRP (mg/L)^a^	< 5	13.0 (7.0–22.0)	8.0 (5.0–21.0)	0.134^c^
LDH (U/L)^a^	110–290	333.0 (281.0–381.0)	321.0 (260.0–367.0)	0.197^c^
ALT (U/L)^a^	5–40	14.0 (11.0–18.0)	14.0 (11.0–18.0)	0.401^c^
PT (s)^b^	9.8–12.1	12.2 (1.1)	12.1 (0.9)	0.535^d^
APTT (s)^b^	21.1–36.5	32.6 (5.4)	32.3 (4.8)	0.721^d^
Fibrinogen (g/L)^a^	1.8–3.5	4.3 (4.0–4.7)	3.9 (2.9–5.4)	0.188^c^
D-Dimer (mg/L)^a^	< 0.5	0.5 (0.4–0.8)	0.4 (0.2–0.8)	0.006^c^
*Lymphocyte subsets*				
CD3 + (%)^a^	61.0–71.8	69.9 (63.2–73.8)	68.2 (58.6–74.6)	0.872^c^
CD3 + CD4 + (%)^a^	29.8–39.9	36.1 (32.7–40.3)	35.5 (30.7–40.9)	0.424^c^
CD3 + CD8 + (%)^b^	20.4–32.8	26.4 (6.1)	25.7 (7.9)	0.489^d^
CD3 + CD4 + /CD3 + CD8 + ^a^	0.9–1.9	1.4 (1.1–1.6)	1.4 (1.1–1.9)	0.895^c^
CD3-CD19 + (%)^b^	14.4–22.7	16.6 (6.1)	21.4 (10.7)	< 0.001^d^
CD3-CD16 + 56 + (%)^a^	8.4–18.2	12.4 (9.4–18.2)	8.4 (5.9–13.1)	< 0.001^c^
CD3 + count (× 10⁹/L)^a^	1.4–3.4	1.3 (1.0–1.7)	2.0 (1.5–2.5)	< 0.001^c^
CD4 + count (× 10⁹/L)^a^	0.7–1.8	0.7 (0.5–0.9)	1.0 (0.7–1.3)	< 0.001^c^
CD8 + count (× 10⁹/L)^a^	0.5–1.4	0.5 (0.4–0.7)	0.7 (0.5–1.0)	< 0.001^c^
*Immunoglobulins*				
IgA (g/L)^a^	0.2–3.2	1.4 (1.0–1.8)	1,0 (0.8–1.6)	0.001^c^
IgG (g/L)^b^	4–16	10.1 (2.3)	9.8 (2.9)	0.506^d^
IgM (g/L)^a^	0.4–2.4	1.2 (0.9–1.6)	1.4 (1.1–1.8)	0.056^c^
IgE (IU/mL)^a^	< 60	93.8 (30.9–311.5)	158.0 (60.4–427.0)	0.065^c^
*Cytokines in BALF*				
IL-1β (pg/mL)^a^	≤ 12.4	1251.6 (429.1–3243.1)	68.9 (27.0–192.4)	< 0.001^c^
IL-5 (pg/mL)^a^	≤ 3.1	3.8 (2.5–19.0)	2.5 (2.4–5.6)	0.032^c^
IL-6 (pg/mL)^a^	≤ 5.4	282.4 (79.5–724.7)	44.9 (13.3–122.8)	< 0.001^c^
IL-8 (pg/mL)^a^	≤ 20.6	2465.9 (1429.9–5419.6)	963.1 (548.1–2288.6)	< 0.001^c^
IL-10 (pg/mL)^a^	≤ 12.9	13.3 (2.6–52.9)	2.4 (2.4–2.4)	< 0.001^c^
IL-17 (pg/mL)^a^	≤ 21.4	20.9 (2.4–42.0)	11.7 (2.4–35.3)	0.176^c^
IFN-γ (pg/mL)^a^	≤ 23.1	22.4 (5.5–125.5)	3.6 (2.5–10.9)	< 0.001^c^
TNF-α (pg/mL)^a^	≤ 16.5	27.8 (4.2–107.1)	3.5 (2.4–5.8)	< 0.001^c^
*BALF cytology*				
BALF WBC (× 10⁶/L)^a^	< 500	2880.0 (1412.3–5030.0)	604.0 (164.0–1820.0)	< 0.001^c^
Neutrophil (%)^a^	≤ 3	70.0 (58.5–80.0)	46.0 (33.0–68.0)	< 0.001^c^
Lymphocyte (%)^a^	≤ 15	10.0 (6.0–18.8)	15.0 (10.0–23.0)	0.011^c^
Macrophage (%)^a^	> 85	12.0 (6.0–19.0)	22.0 (12.0–41.3)	< 0.001^c^
Monocyte (%)^a^	< 5	2.0 (0.0–10.0)	0.0 (0.0–5.3)	0.124^c^

Values expressed as ^a^median (IQR), ^b^mean (SD)

WBC, white blood cell; PLT, platelets; PCT, procalcitonin; ESR, erythrocyte sedimentation rate; CRP, C-reactive protein; LDH, lactate dehydrogenase; ALT, alanine aminotransferase; PT, prothrombin; APTT, activated partial thromboplastin time

^c^Mann-Whitney U Test, ^d^independent samples t-test, ^e^Pearson Chi square Test

Within the MRMP group, patients were stratified according to BALF MP-DNA load (> 10⁶ vs ≤ 10⁶ copies/mL). Those with high MP-DNA loads had significantly longer hospital stay and higher alanine aminotransferase (ALT) levels, as well as higher BALF cytokine levels (IL-1β, IL-6, IL-8, IL-10, IFN-γ, TNF-α), compared with those with low loads. Other clinical features and most peripheral blood markers showed no significant differences between the two subgroups (Table 2). These findings suggest that pathogen load is associated not only with local airway inflammation but also with systemic disease burden.

**Table 2:** Clinical characteristics and laboratory findings of high and low MP-DNA load groups in MRMP patients

Variable	Normal range	High MP-DNA MPP group (n = 61)	Low MP-DNA MPP group (n = 78)	*P value*
*Demographics and clinical features*				
Age, years^a^	–	7.0 (4.0–9.0)	7.0 (5.0–8.0)	0.910^c^
Male, n (%)	–	28 (45.9)	41 (52.6)	0.436^e^
Length of hospital stay, days^a^	–	7 (5–8)	5 (4–7)	0.006^c^
Fever duration, days^a^	–	8 (6–9)	7 (5–8)	0.287^c^
*Peripheral blood markers*				
WBC (*10⁹/L)^a^	4.0–10.0	6.35 (5.20–7.59)	6.27 (5.19–7.46)	0.749^c^
PLT (*10⁹/L)^a^	100–300	255.00 (219.00–334.50)	272.00 (229.25–319.25)	0.590^c^
PCT (ng/mL)^a^	< 0.1	0.09 (0.03–0.25)	0.15 (0.06–0.25)	0.225^c^
ESR (mm/h)^a^	0–20	42.0 (24.5–66.5)	39.0 (29.0–57.3)	0.636^c^
CRP (mg/L)^a^	< 5	13.0 (7.5–20.5)	12.5 (7.0–22.0)	0.873^c^
LDH (U/L)^a^	110–290	338.0 (292.0–421.0)	322.0 (274.8–370.0)	0.102^c^
ALT (U/L)^a^	5–40	15.0 (12.0–21.5)	13.0 (11.0–16.3)	0.029^c^
PT (s)^b^	9.8–12.1	12.1 (1.1)	12.3 (1.1)	0.387^d^
APTT (s)^b^	21.1–36.5	31.7 (5.00)	33.3 (5.6)	0.068^d^
Fibrinogen (g/L)^a^	1.8–3.5	4.2 (4.0–4.9)	4.3 (4.0–4.7)	0.680^c^
D-Dimer (mg/L)^a^	< 0.5	0.6 (0.4–0.9)	0.5 (0.3–0.7)	0.305^c^
*Lymphocyte subsets*				
CD3 + (%)^a^	61.0–71.8	67.3 (63.0–73.1)	69.5 (63.4–73.9)	0.830^c^
CD3 + CD4 + (%)^a^	29.8–39.9	36.1 (32.1–40.2)	36.6 (32.8–40.6)	0.200^c^
CD3 + CD8 + (%)^b^	20.4–32.8	26.5 (5.8)	26.4 (6.4)	0.933^d^
CD3 + CD4 + / CD3 + CD8 + ^a^	0.9–1.9	1.4 (1.1–1.6)	1.4 (1.1–1.7)	0.857^c^
CD3-CD19 + (%)^b^	14.4–22.7	17.2 (6.4)	16.2 (5.8)	0.352^d^
CD3-CD16 + 56 + (%)^a^	8.4–18.2	13.6 (9.7–17.8)	11.5 (9.1–18.5)	0.625^c^
CD3 + count (× 10⁹/L)^a^	1.4–3.4	1.2 (0.9–1.7)	1.4 (1.0–1.8)	0.258^c^
CD4 + count (× 10⁹/L)^a^	0.7–1.8	0.6 (0.5–0.9)	0.8 (0.6–0.9)	0.203^c^
CD8 + count (× 10⁹/L)^a^	0.5–1.4	0.5 (0.3–0.7)	0.5 (0.4–0.7)	0.517^c^
*Immunoglobulins*				
IgA (g/L)^a^	0.2–3.2	1.4 (1.0–1.8)	1.3 (1.0–1.9)	0.967^c^
IgG (g/L)^b^	4–16	10.0 (2.3)	10.1 (2.3)	0.802^d^
IgM (g/L)^a^	0.4–2.4	1.1 (0.9–1.5)	1.2 (0.9–1.6)	0.397^c^
IgE (IU/mL)^a^	< 60	81.4 (23.6–231.0)	120.0 (47.4–368.2)	0.058^c^
*Cytokines in BALF*				
IL-1β (pg/mL)^a^	≤ 12.4	2212.5 (1312.4–5425.0)	835.8 (158.7–1641.3)	< 0.001^c^
IL-5 (pg/mL)^a^	≤ 3.1	4.0 (2.4–23.1)	3.6 (2.4–11.1)	0.172^c^
IL-6 (pg/mL)^a^	≤ 5.4	471.5 (167.9–1355.3)	202.4 (48.5–479.3)	< 0.001^c^
IL-8 (pg/mL)^a^	≤ 20.6	2903.0 (1987.1–7284.1)	1735.4 (906.3–4211.8)	< 0.001^c^
IL-10 (pg/mL)^a^	≤ 12.9	24.8 (4.8–96.7)	9.6 (2.4–37.9)	0.010^c^
IL-17 (pg/mL)^a^	≤ 21.4	22.1 (3.4–41.3)	20.0 (2.4–43.0)	0.841^c^
IFN-γ (pg/mL)^a^	≤ 23.1	44.6 (8.4–185.0)	15.9 (4.3–72.8)	< 0.017^c^
TNF-α (pg/mL)^a^	≤ 16.5	60.4 (6.6–202.2)	23.2 (3.2–65.8)	< 0.015^c^
*BALF cytology*				
BALF WBC (*10⁶/L)^a^	< 500	2961 (1458–5239)	2880 (1200–4511)	0.296^c^
Neutrophil (%)^a^	≤ 3	70 (60–80)	70 (51–80)	0.686^c^
Lymphocyte (%)^a^	≤ 15	10.0 (6.0–18.0)	12.0 (6.5–19.5)	0.385^c^
Macrophage (%)^a^	> 85	9 (4–19)	15 (7–19)	0.108^c^
Monocyte (%)^a^	< 5	2.5 (0.0–10.0)	2.5 (0.0–10.0)	0.839^c^

Spearman’s correlation analysis confirmed that BALF MP-DNA load positively correlated with IL-1β (r = 0.61, *P* < 0.001) and IL-6 (r = 0.48, *P* < 0.001), indicating that higher pathogen burden is associated with an enhanced local inflammatory response.

## Discussion

This study showed that macrolide resistance in pediatric MPP was associated with prolonged fever, longer duration of hospitalization, altered immune cell profiles, and enhanced airway inflammation compared with macrolide-sensitive infection [10, 11]. In MRMP cases, higher BALF MP-DNA load was linked to stronger local cytokine responses and higher liver enzyme levels. Importantly, we observed positive correlations between BALF IL-1β and IL-6 levels and MP-DNA load, providing new insight into how pathogen burden influences local immune activation in pediatric MPP.

These observations are supported by previous studies [12, 13]. These findings suggest that cytokine production is closely linked to pathogen activity, as MP-DNA copy number reflects the replicative status of the organism. Wang, et al. demonstrated that serum IL-6 levels can be useful predictors for severe MPP [14]. Fan, et al. reported significantly higher serum IL-1β levels in refractory MPP compared to general MPP [15]. The current study extends these results by showing a positive correlation between BALF IL-1β and MP-DNA load, highlighting the value of local cytokine measurement, which has been rarely reported. In addition, serum IL-13 and IL-33 levels were reported to be significantly higher in MRMP-infected patients than in those infected with macrolide-sensitive strains, supporting the idea that antibiotic resistance may influence host immune responses [16].

The clinical implications of this study deserve attention. In our hospital, mutation analysis of the 23S rRNA gene is routinely performed on BALF samples, and results are typically available within two days. Clinicians reviewed these reports in real time and adjusted antibiotic regimens when macrolide resistance was detected, most often switching to second-line agents such as minocycline or fluoroquinolones in accordance with pediatric guidelines. These real-time molecular results allow for antibiotic adjustment which explains the reduced disease severity despite macrolide resistance. This rapid turnaround and integration of molecular results into clinical practice may have contributed to improved treatment precision and highlights the importance of incorporating resistance testing into routine management of pediatric MPP. D-dimer elevation was also observed in MRMP patients, consistent with previous reports identifying D-dimer as a marker associated with MRMP infection [17]. These findings together indicate that resistance and pathogen burden shape distinct immune–inflammatory patterns, which may explain the prolonged fever and hospital stay observed in MRMP cases and could have clinical value for risk stratification and treatment decisions.

The strengths include the relatively large pediatric cohort undergoing bronchoscopy, simultaneous evaluation of systemic and local immune-inflammatory responses, and integration of resistance genotyping with quantitative bacterial load assessment. However, it was a single-center retrospective study, and bronchoscopy was mainly performed in children with more severe disease. Being an observational study, causal relationships cannot be established.

In conclusion, MRMP and high BALF MP-DNA load are associated with enhanced airway inflammation and prolonged clinical course in pediatric MPP. The observed correlation between BALF IL-1β and pathogen load adds to current knowledge and suggests that local airway cytokine assessment may help evaluate disease severity. Prospective multicenter studies are warranted to validate these findings and to determine whether early targeted interventions can improve outcomes.

## What This Study Adds?

- Macrolide resistance in pediatric MPP was associated with prolonged fever, longer duration of hospitalization, altered immune cell profiles, and enhanced airway inflammation.
- Higher pathogen burden was associated with an enhanced local inflammatory response.

## Data Availability

The data that support the findings of this study are available from the corresponding author upon reasonable request.
